# Multiple sclerosis and intracellular cobalamin defect (*MMACHC*/*PRDX1*) comorbidity in a young male

**DOI:** 10.1016/j.ymgmr.2019.100560

**Published:** 2020-01-07

**Authors:** Luca Pollini, Manuela Tolve, Francesca Nardecchia, Serena Galosi, Claudia Carducci, Emanuele di Carlo, Carla Carducci, Vincenzo Leuzzi

**Affiliations:** Sapienza University of Rome, Department of Human Neuroscience, Rome, Italy; Sapienza University of Rome, Department of Experimental Medicine, Rome, Italy

**Keywords:** Multiple sclerosis, cblC, PRDX1, MMACHC, cblC, cobalamin deficiency type C, homocysteine, hcy, methylmalonic acid, MMA, multiple sclerosis, MS, magnetic resonance imaging, MRI

## Abstract

**Background:**

Methylmalonic acidaemia with homocystinuria type C (cblC defect) is an inherited error of cobalamin metabolism. Cobalamin deficient processing results in high levels of methylmalonic acid and homocysteine. The latter is considered to be a risk factor for multiple sclerosis (MS). We report on the first case of a patient with comorbid cblC defect and MS.

**Case report:**

This young male presented at the age of 14 with a relapsing-remitting neurological disorder associated with imaging alterations suggestive of MS. Treatment resulted in a partial clinical improvement with vanishing of white matter lesions. Later on, the emergence of unexpected clinical features led to a metabolic work-up, revealing a cobalamin intracellular defect. Genetic analysis disclosed a single variant in *MMACHC* (c.482G > A; p.Arg161Gln) and another splicing variant in *PRDX1* (c.1-515G > T) that cause the silencing of the wild-type *MMACHC* allele, so confirming the diagnosis of cblC defect. Although cblC treatment was effective, when 17-year-old he experienced a relapse of neurological symptoms. Further imaging and laboratory studies eventually supported the diagnosis of MS.

**Discussion:**

While the comorbid association of MS and cblC in our patient may remain anecdotic, we suggest measuring Hcy and MMA levels in young patients with a relapsing-remitting demyelinating disorder, in order not to miss a cblC defect, that requires a specific and effective treatment.

## 1 Background

Methylmalonic acidemia with homocystinuria, cobalamin deficiency type C (cblC) is a rare disorder of intracellular cobalamin metabolism, characterized by neurological, cardiac, renal, haematological, and ophtalmological impairment. While the majority of patients become symptomatic during the first year of life, adolescents and adult patients mainly present with psychiatric symptoms, dementia, myelopathy, peripheral neuropathy and thrombosis [1]. High levels of homocysteine (Hcy) and methylmalonic acid (MMA) are the biochemical markers of the disease. A relapsing-remitting neurological deterioration, a typical presentation of multiple sclerosis (MS), has been reported in cblC patients [2,3]. Although in the largest cohort of patients with cobalamin-dependent remethylation disorders the association with MS has not been observed [4], a mild increase of Hcy (ranging between 20 and 30 μmol/L) is regarded as a risk factor for MS [5]. The comorbid occurrence of both conditions in a young patient is the object of the present report.

## 2 Case report

This 17-year-old male is the first child of nonconsanguineous healthy parents. His younger sister is healthy. He was born after a pregnancy complicated by rubeola infection: at birth anophthalmia of the right eye was detected and brain imaging showed a congenital arachnoid cyst in the left temporal lobe. Familial history revealed a father's 1st cousin diagnosed with MS in adulthood. When 14-year-old, the patient complained a decline in school performance and a loss of strength of the inferior limbs. A year later, he was admitted to a Neurology Unit due to sudden visual loss (3/10 in the left eye) and the subacute emergence of a spastic paraparesis. A brain magnetic resonance imaging (MRI) showed multiple subcortical and periventricular white matter lesions suggesting the diagnosis of MS. The treatment with steroid improved visual acuity (8/10), but it was ineffective on lower limb spasticity. Subsequently, a treatment with interferon 1-b was started. On follow-up, a brain MRI showed a complete vanishing of the white matter lesions. However, at the age of 16, he suffered from a sudden episode of lethargy, loss of ambulation, bilateral upper limb dystonia and urinary incontinence. Brain and spinal MRI were normal. A partial spontaneous recovery was observed during the following weeks. A comprehensive clinical evaluation revealed a hypertrophic cardiomyopathy and proteinuria. A few months later, on examination he showed spastic paraparesis, pale optic disc, and large-amplitude horizontal left beating nystagmus. A further metabolic work-up revealed high Hcy (162 μmol/L; normal 4.7–11.3 μmol/L) and low methionine (23 μmol/L; normal 26–38 μmol/L) plasmatic levels and a marked increase of urinary MMA (1075 mmol/mol creatinine; normal 0.4–23 mmol/mol creatinine). Molecular analysis of *MMACHC* gene (NM_015506) revealed a heterozygous variant in exon 4 (c.482G > A; p.Arg161Gln), inherited from his father. The lack of other pathogenetic variants on the *MMACHC* gene prompted us to sequence the *PRDX1* gene (NM_001202431) that revealed a c.515-1G > T splicing variant, inherited from the mother, and confirmed the diagnosis of cobalamin disorder type C [6].

**Fig. 1:**
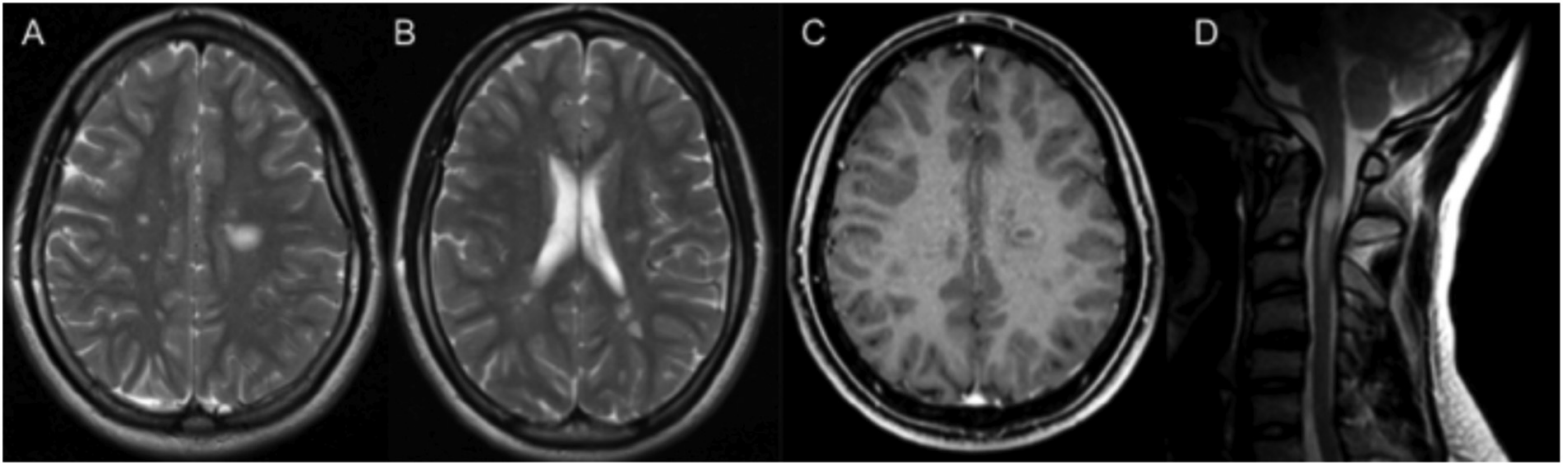
Brain and spinal MRI of the patient at the age of 17 (A) Note the disseminate white matter lesions on the axial T2-weighted sequence. (B) Lower section shows more asymmetrical and multifocal hyperintense lesions of white matter. (C) The large left white matter lesion shows and open-ring enhancement on the axial T1-weighted contrast sequence. (D) T2-weighted image of the cervical spine shows an hyperintense lesions at C2-C3 level.

The treatment with betaine (6 g/day), carnitine (6 g/day), hydroxy-cobalamin (5000 μg on alternate days), and folinic acid (15 mg/day) resulted in a remarkable lowering of plasma Hcy (27 μmol/L) and urine MMA (47 mmol/mol creatinine) as well as in an improvement of spastic paresis with restoration of autonomous gait. Unexpectedly, at the age of 17, he complained an acute loss of strength and paresthesia in the right arm. A brain and spinal MRI showed asymmetrical and multifocal cerebral white matter lesions, some of them with an open-ring enhancement, and a C2-C3 medullary lesion (Fig. 1). Oligoclonal bands were detected in CSF and HLA-study showed DRB1*13–15 haplotype. The diagnosis of relapsing-remitting MS was definitively made in comorbidity with cblC defect. The patient has received treatment with natalizumab for 10 months in addition to the specific treatment for cblC. During the follow-up we observed no relapses nor novel lesions at MRI and no progression of cardiac nor renal dysfunctions. Written informed consent of patient and his parents was obtained.

## 3 Discussion

The patient here reported suffered from a relapsing-remitting demyelinating disorder resulting from an early onset MS and a concomitant alteration of cobalamin metabolism. While he showed a complete remission of white matter lesions under steroid treatment, which is a relatively common feature in pediatric-onset MS [7], other neurological deficits, and the renal and cardiac involvement pointed to the diagnosis of cblC defect. Interestingly, a number of patients with cblC and cblG defect presented with relapsing-remitting neurological symptoms, elevation of intrathecal IgG or protein in some of them [9] (but not the presence of oligoclonal bands), and an unusual responsiveness to steroid treatment, that were initially misdiagnosed as MS [2,3,8,9]. The longest follow-up of a patient with a late onset cblC reported a persistent improvement of the patient's clinical status after the start of the cblC treatment. In contrast, our patient, despite an excellent metabolic control, experienced a further relapse of neurological symptoms, the appearance of novel white matter lesions on MRI, and the presence of oligoclonal bands in CSF, suggesting a comorbid condition rather than a phenocopy of MS.

*MMACHC* gene product is involved in the regeneration of S-adenosylmethionine, the most important methyl donor, from Hcy. Several MS risk loci have been found in this metabolic pathway, such as *SCL19A1*, *SHMT*, *MTHFR*, *CBS*, suggesting a major role of methylation processes in MS pathogenesis [10]. Recently, c.515-1G > T splicing variant in *MMACHC* adjacent gene *PRDX1*, was demonstrated to cause *MMACHC* gene silencing by methylation of the promoter through a mechanism called “*epi-cblc”* [6]. The present case is the ninth patient with cblC caused by this complex pathogenic interaction. Interestingly: a) *PRDX1* encodes the peroxiredoxin-1, an enzyme induced by oxidative stress to protect the integrity of the blood brain barrier; and b) vascular peroxiredoxin-1 immunoreactivity is upregulated in active MS lesions in brain tissues [11]. So far MMA has not been associated with an increased risk of MS [12].

While the comorbid association of MS and cblC in our patient may remain an anecdotic observation, given the higher prevalence of MS than intracellular cobalamin defects in this age group, we suggest measuring Hcy and MMA levels in young patients with a relapsing-remitting demyelinating disorder, in order not to miss a cblC defect, that requires a specific and effective treatment.

## Financial disclosures

None of the authors have any disclosure to declare.

## Data availability statement

Data availability not applicable.

## Editorial policies and ethical consideration

All the reported investigations and a specific permission for the publication of the results was obtained through a written informed parental consent.
